# The drug efflux pump Pgp1 in pro-inflammatory lymphocytes is a target for novel treatment strategies in COPD

**DOI:** 10.1186/1465-9921-14-63

**Published:** 2013-06-03

**Authors:** Greg Hodge, Mark Holmes, Hubertus Jersmann, Paul N Reynolds, Sandra Hodge

**Affiliations:** 1 Lung Research, Hanson Institute and Department of Thoracic Medicine, Royal Adelaide Hospital, Adelaide, South Australia; 2 Department of Medicine, University of Adelaide, Adelaide, South Australia

**Keywords:** COPD, Pgp1, Pro-inflammatory lymphocytes, Granzyme B

## Abstract

**Background:**

Pro-inflammatory/cytotoxic T cells (IFNγ, TNFα, granzyme B+) are increased in the peripheral circulation in COPD. NKT-like and NK cells are effector lymphocytes that we have also shown to be major sources of pro-inflammatory cytokines and granzymes. P-glycoprotein 1 (Pgp1) is a transmembrane efflux pump well characterised in drug resistant cancer cells. We hypothesized that Pgp1 would be increased in peripheral blood T, NKT-like and NK cells in patients with COPD, and that this would be accompanied by increased expression of IFNγ, TNFα and granzyme B. We further hypothesized that treatment with cyclosporine A, a Pgp1 inhibitor, would render cells more sensitive to treatment with corticosteroids.

**Methods:**

Pgp1, granzyme B, IFNγ and TNFα expression were measured in peripheral blood T, NK and NKT-like cells from COPD patients and control subjects (± cyclosporine A and prednisolone) following *in vitro* stimulation and results correlated with uptake of efflux dye Calcein-AM using flow cytometry.

**Results:**

There was increased Pgp1 expression by peripheral blood T, NKT-like and NK cells co-expressing IFNγ, TNFα and granzyme B in COPD patients compared with controls (e.g. %IFNγ/Pgp1 T, NKT-like, NK for COPD (Control): 25(6), 54(27), 39(23)). There was an inverse correlation between Pgp1 expression and Calcein-AM uptake. Treatment with 2.5 ng/ml cylosporin A and10^-6^ M prednisolone resulted in synergistic inhibition of pro-inflammatory cytokines in Pgp1 + cells (p < 0.05 for all).

**Conclusions:**

Treatment strategies that target Pgp1 in T, NKT-like and NK cells may reduce systemic inflammatory mediators in COPD and improve patient morbidity.

## Background

COPD is predicted to be the 3rd leading cause of death worldwide by 2020 [1]. Existing treatments are largely symptomatic and the only approved anti-inflammatory medication, corticosteroids, has no proven disease modifying effect [1]. Inhaled corticosteroids have major benefits for the treatment of airway inflammation in asthma, but the reason for their relative lack of efficacy in COPD is both poorly understood and a major limiting factor in COPD treatment. Thus, better understanding of the mechanisms underlying steroid resistance in COPD, and a way to circumvent this to take better advantage of existing therapies would have an immediate clinical impact.

COPD is a systemic disease and may represent a “spill-over” of inflammatory events occurring in the lungs [2]. In this regard we have previously shown an increase in pro-inflammatory/cytotoxic T cells, NKT-like and NK cells in the peripheral blood and airways in COPD patients compared with non-COPD smokers where some changes were only noted in the lungs compared with healthy controls [3-5].

P-glycoprotein 1 (Pgp1) is a transmembrane efflux pump well characterised in drug resistant cancer cells [6]. We hypothesized that Pgp1 may play a role in steroid resistance and would be increased in peripheral blood T, NKT-like and NK cells in patients with COPD, and that this would be accompanied by increased expression of IFNγ, TNFα and granzyme B. We further hypothesized that treatment with low dose cyclosporine A, a Pgp1 inhibitor, would render cells more sensitive to treatment with corticosteroids.

Pgp1, granzyme B, IFNγ and TNFα expression were measured in peripheral blood T, NK and NKT-like cells from COPD patients and control subjects (± cyclosporine A and prednisolone) following *in vitro* stimulation and results correlated with uptake of efflux dye calcein AM using flow cytometry.

## Methods

### Patient and control groups

COPD patients and controls were recruited for the study and fully informed consent obtained. There was no exacerbation of COPD for 6 weeks prior to involvement in the study. Ethics approval was obtained from the Royal Adelaide Hospital. The diagnosis of moderate COPD was established using the GOLD criteria [7] of a relevant history and post bronchodilator FEV1 30-80% of predicted and FEV1/FVC < 70%.

Blood was collected from 10 patients with COPD (Table 1) of whom all were ex-smokers (at least one year).

**Table 1:** Demographic details of the COPD and control subjects

**Subjects**	**Controls**	**COPD**
No. of subjects	14	10
Age (years)	56 (± 8)	58 (± 16)
FEV1, % pred	110.4 (± 9)	60.5 (± 20)
FEV1, % FVC	96 (± 12)	58 (± 15)*
Male/Female	8/6	6/4

Data shown as mean ± SEM.

Abbreviations: *COPD*: chronic obstructive pulmonary disease. *FEV1*: forced expiratory volume in 1 second; *FVC*: forced vital capacity; *P < 0.05 compared to controls.

Blood was also obtained from 14 non-smoking volunteers (Table 1) with no history of airways disease and normal lung function).

### Leucocyte counts

Full blood counts, including white cell differential counts, were determined on blood specimens using a CELL-DYN 4000 (Abbot Diagnostics, Sydney, Australia). Blood films were stained by the May-Grunwald-Giemsa method and white cell differential counts checked by morphological assessment microscopically.

### CD3, CD4 and CD8 cell counts

The percentages of CD3, CD4 and CD8 lymphocytes were calculated using flow cytometry. One hundred microlitre of peripheral blood were stained with appropriately diluted fluorescently conjugated monoclonal antibodies as previously described [3].

### Granzyme B expression by T, NKT-like and NK cells

The percentages of T, NKT-like and NK cells expressing granzyme B, was determined as previously reported [5].

### Leucocyte stimulation

Leucocyte stimulation was required for both intracellular cytokine and Pgp1 expression by T, NKT-like and NK cells. One mL aliquots of blood (diluted 1:2 with RPMI 1640 medium) were placed in a 10 mL sterile conical PVC tubes (Johns Professional Products, Sydney, Australia). Phorbol myristate (25 ng/mL) (Sigma, Sydney, Australia) and ionomycin (1 μg/mL) (Sigma) was added. Brefeldin A (10 μg/mL) was added as a “Golgi block” (Sigma) and the tubes re-incubated in a humidified 5% CO_2_/95% air atmosphere at 37°C for 16 h.

### Intracellular IFNγ and TNFα expression by T, NKT-like and NK cells

Three hundred and fifty μL of stimulated peripheral blood cells were stained with appropriately diluted fluorescently conjugated monoclonal antibodies as previously reported [3-5] to IFNγ FITC (BD Biosciences, Sydney, Australia) (BD), CD3 perCP.Cy5.5 (BD), CD56 APC (Beckman Coulter, Sydney, Australia), TNFα V450, granzyme B V450 and CD45 V500 (BD). Samples were analysed by gating using forward scatter (FSC) versus side scatter (SSC) to exclude platelets and debris. Gated cells were analysed with CD45 V500 (BD) to ascertain that cells were of lymphoid origin. A minimum of 500,000 CD45 positive, low SSC events were acquired on a FACSCanto II (BD) in list-mode format for analysis using FACSDiva software (BD). T cells were identified as events that were CD3 + CD56-, NK cells as CD3-CD56+ and NKT-like cells as CD3 + CD56+ events as previously reported [5].

### Pgp1 expression by T, NK and NKT-like cells

Preliminary experiments showed that cells required stimulation for significant Pgp1 molecule expression by T, NKT-like and NK cells. Following stimulation as described above, 350 μL aliquots of cells were treated with 2 mL FACSLyse for 10 min. Cells were centrifuged, supernatant discarded and 500 mL FACSPerm added for 10 min. Two mL 0 · 5% bovine serum albumin (BSA) (Sigma) in IsoFlow (Beckman Coulter) was then added and the tubes centrifuged at 300 *g* for 5 min. After decanting supernatant, Fc receptors were blocked with 10 mL human immunoglobulin (Intragam, CSL, Melbourne, Australia) for 10 min at room temperature. Five μL of appropriately diluted CD3 perCP.Cy5.5 (BD), Pgp1 PE (BD) CD56 APC (Beckman Coulter) and CD45 V500 (BD) or isotype control (BD) were added for 15 min in the dark at room temperature. Cells were washed and events acquired and analyzed as described above.

### Pgp1, IFNγ, TNFα and granzyme B expression by T, NKT-like and NK cells

To determine possible association of pro-inflammatory cytokines and granzyme B expression with Pgp1 expression by T, NKT-like and NK cells, whole blood was stimulated as described above. Following stimulation and processing, 5 μL of appropriately diluted IFNγ FITC (BD), granzyme B FITC (BD), Pgp1 PE (BD), CD3 perCP.Cy5.5 (BD), TNFα V450 (BD) and CD45 V500 (BD) were added for 15 min in the dark at room temperature. Cells were washed and events acquired and analyzed as described above.

### Uptake of Calcein-AM by T, NKT-like and NK cells

To determine functional Pgp1 activity, efflux of Calcein-AM was analysed in T, NKT-like and NK cells as previously published [8] from a cohort of COPD patients and control subjects. Briefly, following stimulation of cells as described above, 5 nM Calcein-AM (eBioscience, San Diego, CA, USA) was added and cells re-incubated in a humidified 5% CO_2_/95% air atmosphere at 37°C for 30 min. Aliquots were washed twice with wash buffer to remove free Calcein-AM and cells processed for Pgp1 expression as described above.

### Effect of methylprednisolone and Cyclosporin A on Pgp1, IFNγ, TNFα and granzyme B expression by T, NKT-like and NK cells

To determine the effects of methylprednisolone and Cyclosporin A on Pgp1, IFNγ, TNFα and granzyme B expression by T, NKT-like and NK cell subsets, one mL aliquots of blood (diluted 1:2 with RPMI 1640 medium) were placed in a 10 mL sterile conical PVC tubes with 10^-6^ M methylprednisolone and/or various concentrations of Cyclosporin A (0, 1, 2.5, 5, 10, 50, 100, 200 and 250 ng/mL) for 24 h in a humidified 5% CO_2_/95% air atmosphere at 37°C. Blood cultures were then stimulated as described above for 16 h and processed for Pgp1, IFNγ, TNFα, granzyme B and perforin expression by T, NKT-like and NK subsets as described above.

### Statistical analysis

Statistical analysis was performed using Mann–Whitney and Spearman Rho correlation tests using SPSS software and differences between groups of P < 0.05 considered significant.

## Results

### Blood CD4+ and CD8+ T cell counts

There was a significant increase in the absolute number of CD8 T cells in blood from COPD patients compared with controls (0.43 ± 0.22 and 0.33 ± 0.16 × 10^9^/L for COPD patients and controls respectively, P = 0.047). There were no other significant differences in the absolute counts of CD3+, CD4+ or CD8+ absolute counts. The percentage of CD8+ T cells was significantly increased and CD4+ T cells significantly decreased in the COPD group compared to the control group (57 ± 8 and 69 ± 7 for CD4 and 43 ± 9 and 31 ± 8 for CD8 for COPD patients and controls respectively, P = 0.032 and P = 0.037 respectively). The percentage of CD4-CD8- and CD4 + CD8+ T cells was < 3% for all patient and control subjects. There was no change in the percentage or absolute numbers of NK and NKT-like cells between COPD patients and control group (p < 0.05 for all) consistent with our previous report [9] (data not shown).

### Pgp1 expression by T, NKT-like and NK cells

**Figure 1:**
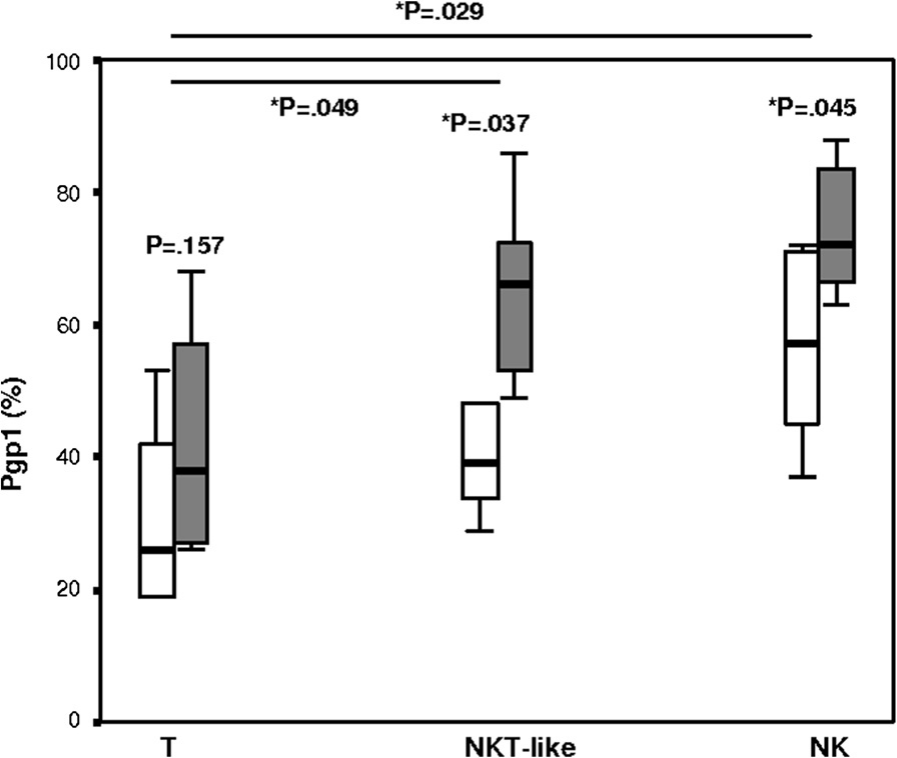
**Pgp1 + T, NKT-like and NK cells in COPD and control subjects.** Data presented as box plots. There was a significant increase in the percentage of Pgp1 + NKT-like and Pgp1 + NKcells in COPD patients (grey bars) compared with control subjects (clear bars). The percentage of Pgp1 + NKT-like and Pgp1 + NK cells was greater than the percentage of Pgp1 + T cells for COPD patients but not control subjects (p > 0.05 for all).

There was no significant differences in Pgp1 expression by stimulated CD3+ T cells (Figure 1). There was however a significant increase in Pgp1 expression by NKT-like and NK cells from COPD patients versus controls (Figure 1). Pgp1 expression was higher in NKT-like and NK cells than T cells for COPD patients but not control subjects (p > 0.05 for all).

### IFNγ, TNFα and granzyme B expression by T, NKT-like and NK cells

**Figure 2:**
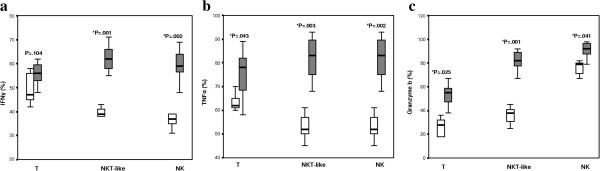
**IFNγ +, TNFα and Granzyme b + T, NKT-like and NK cells in COPD and control subjects.** Data presented as box plots. **a.** There was a significant increase in IFNγ expression by NKT-like and NK cells in COPD patients (grey bars) compared with controls (clear bars) but not T cells. **b**. There was a significant increase in TNFα expression by T, NKT-like and NK cells in COPD patients compared with controls. **c**. There was a significant increase in granzyme b expression by T, NKT-like and NK cells in COPD patients compared with controls.

There was no significant increase in IFNγ expression by stimulated CD3+ T cells from COPD patients versus controls (Figure 2a). There was however a significant increase in IFNγ expression by NKT-like and NK cells in COPD patients compared with controls (Figure 2a).

There was a significant increase in both TNFα and granzyme B expression by CD3+ T, NKT-like and NK cells in COPD patients compared with controls (Figure 2b and 2c).

### IFNγ, TNFα and granzyme B expression by Pgp1+ T, NKT-like and NK cells

**Figure 3:**
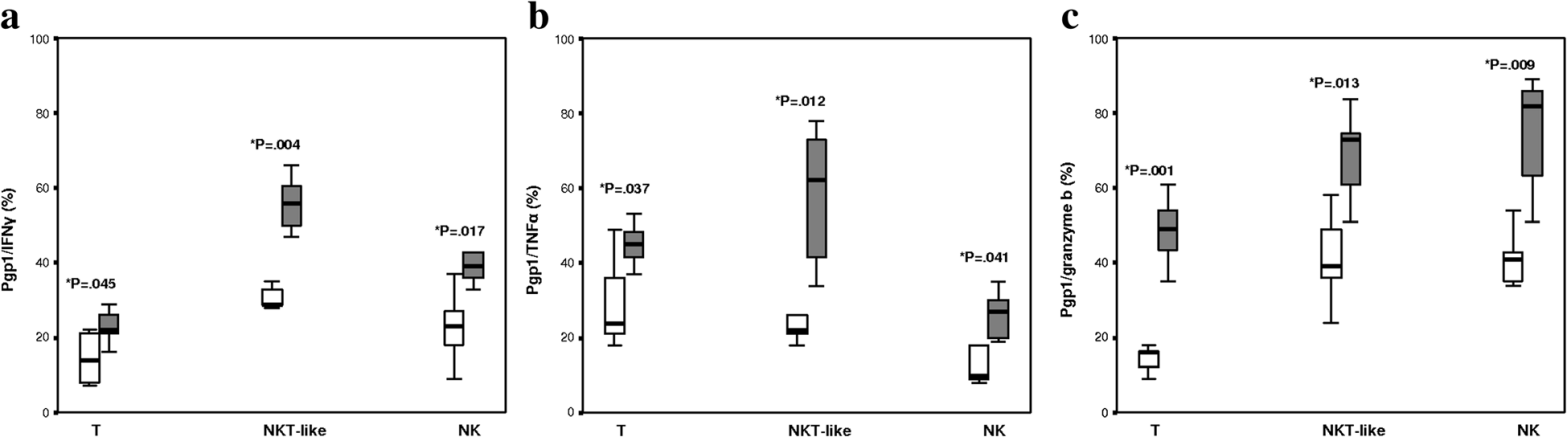
**Pgp1+ T, NKT-like and NK cells producing IFNγ, TNFα and Granzyme b in COPD and control subjects.** Data presented as box plots. **a.** There was a significant increase in IFNγ expression by Pgp1+ T, NKT-like and NK cells in COPD patients compared with controls. **b**. There was a significant increase in TNFα expression by Pgp1+ T, NKT-like and NK cells in COPD patients compared with controls. **c**. There was a significant increase in granzyme b expression by Pgp1+ T, NKT-like and NK cells in COPD patients compared with controls.

**Figure 4:**
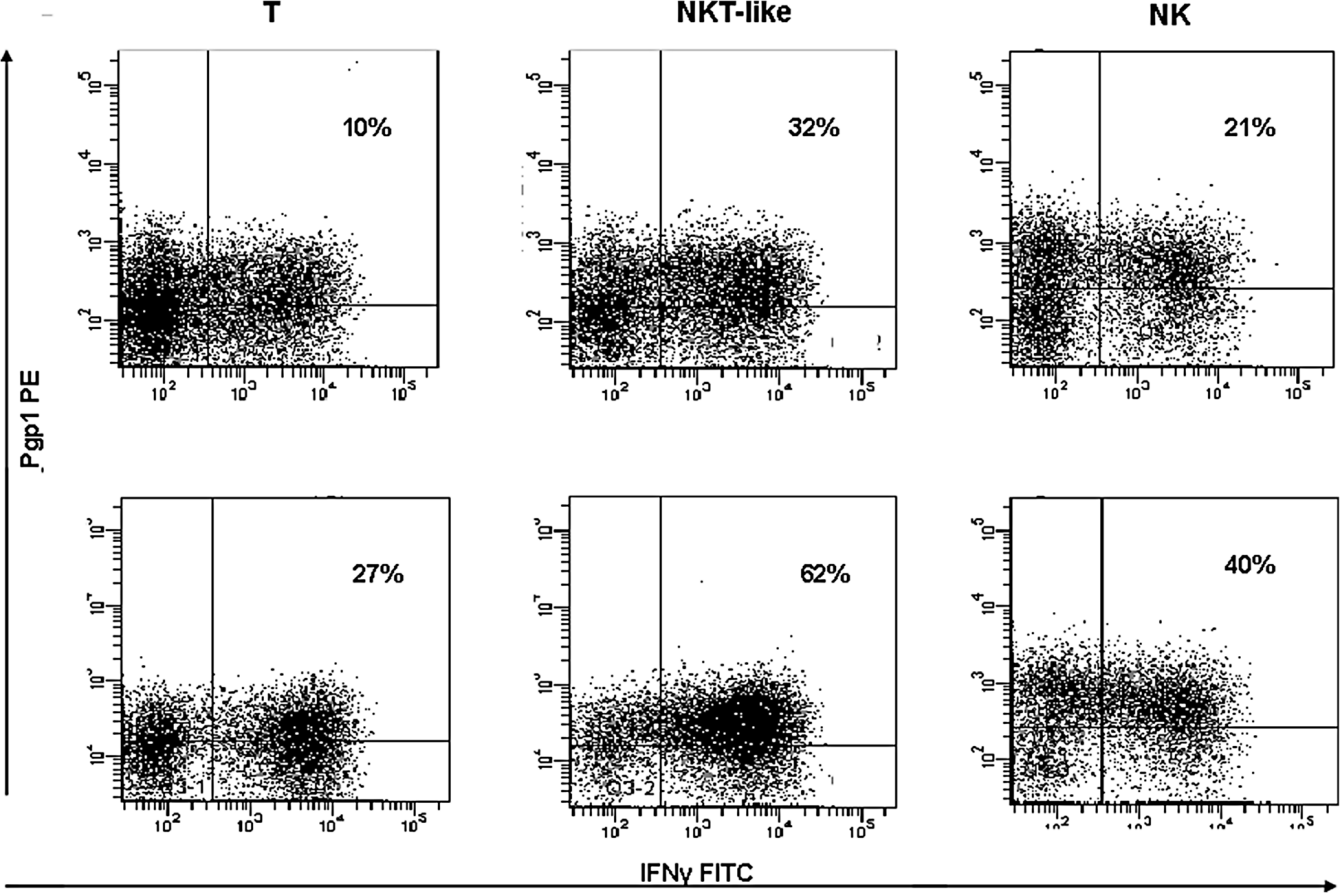
Representative dot plots showing increased IFNγ expression by Pgp1+ T, NKT-like and NK cells from a patient with COPD (bottom row) compared with a control subject (upper row).

There was a significant increase in IFNγ, TNFα and granzyme B expression by Pgp1+ T, NKT-like and NK cells in COPD patients compared with controls (Figure 3a, 3b and 3c). Granzyme B expression was significantly increased in stimulated T, NKT-like and NK cells from both COPD patients and controls compared with non-stimulated blood (p < 0.05 for all) (data not shown). Representative dot plots showing increased IFNγ expression by Pgp1+ T, NKT-like and NK cells from a patient with COPD compared with a control subject are shown in Figure 4.

### Correlation between Pgp-1 expression and Calcein-AM expression by T, NKT-like and NK cells

**Figure 5:**
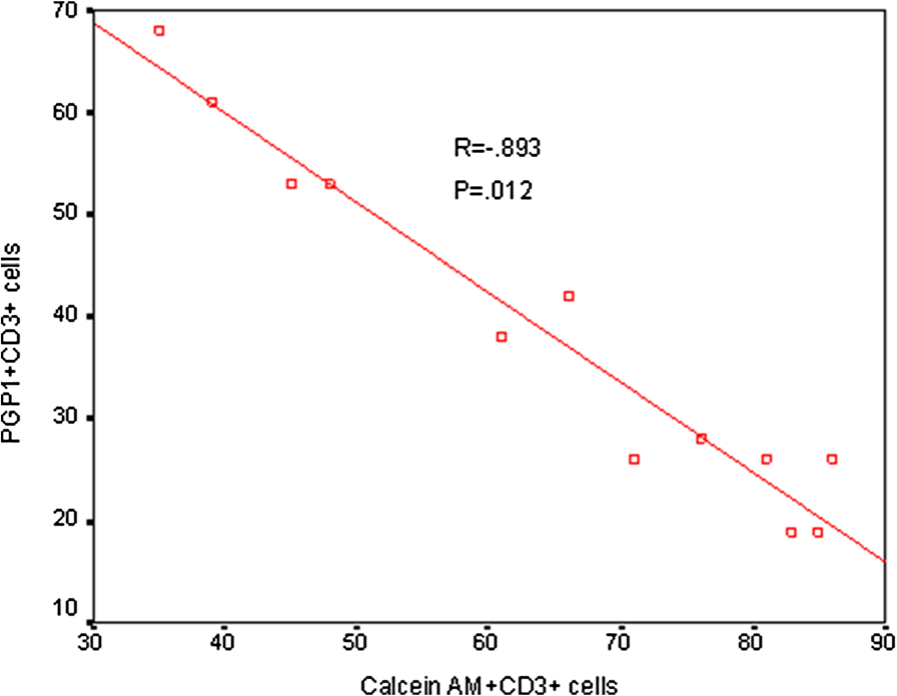
**Correlation between Calcein-AM and Pgp1 in T cells.** Graph of Calcein-AM + CD3+ versus Pgp1 + CD3+ T cells. There was a significant negative correlation between Pgp1 expression and Calcein-AM uptake by CD3 + T cells from a cohort of 6 COPD patients and 6 control subjects.

Calcein-AM has been shown to enter cells that are deficient in Pgp1 expression [8]. There was a significant negative correlation between Pgp1 expression and Calcein-AM uptake by T (R = .893, p = .012) (Figure 5), NKT-like (R = .901, p = .008) and NK cells (R = .915, p = .004) from a cohort of 6 COPD patients and 6 control subjects.

### Effect of methylprednisolone and Cyclosporin A on Pgp1, IFNγ, TNFα and granzyme B expression by T, NKT-like and NK cells

**Figure 6:**
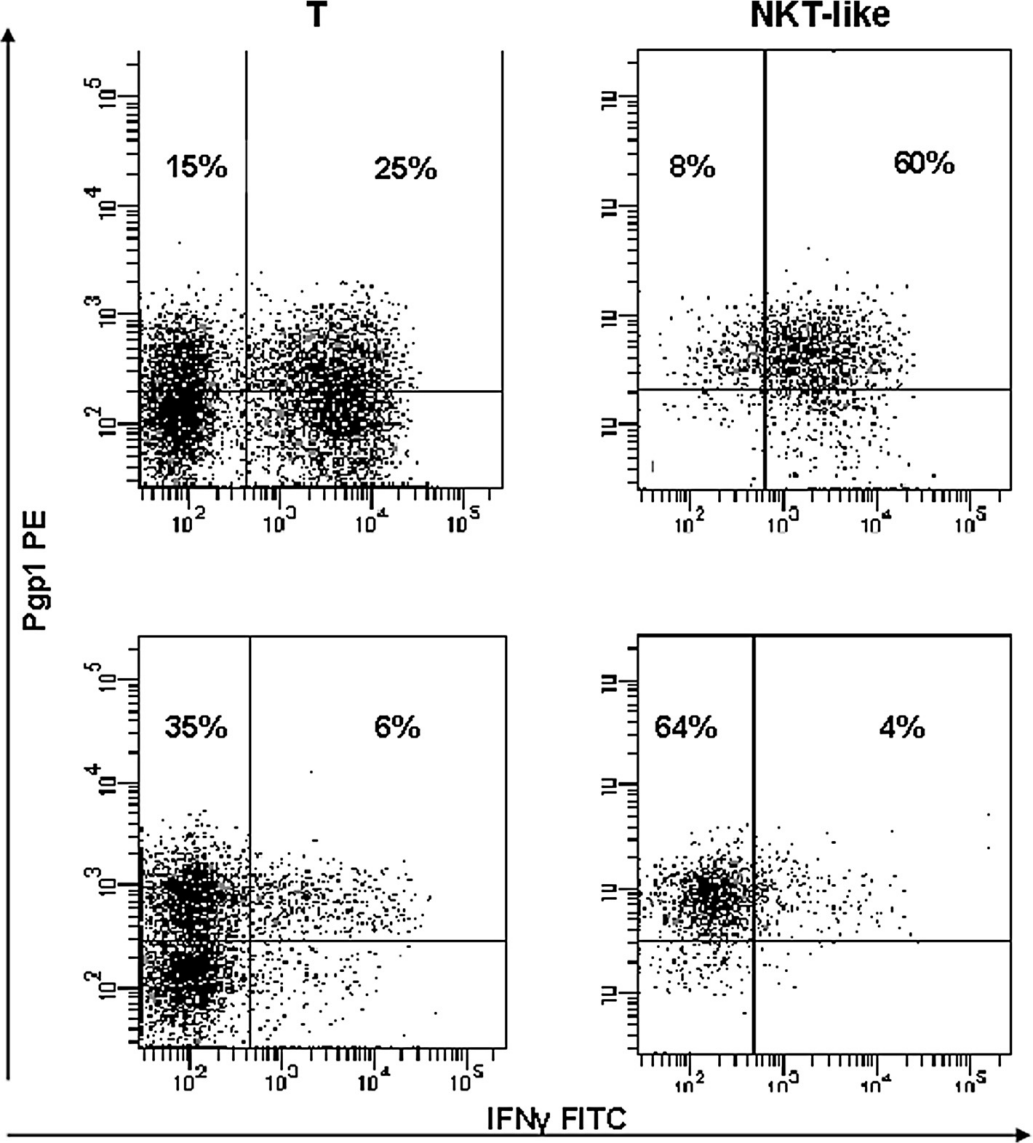
Representative plots showing the combined inhibitory effect of 10^-6^ M methylprednisolone and 2.5 ng/mL Cyclosporin A on IFNγ expression by Pgp1+ T and NKT-like cells (lower plots) compared with media only (top plots).

The inhibitory effect of 10^-6^ M methylprednisolone alone and in combination with 2.5 ng/mL Cyclosporin A on Pgp1, IFNγ, TNFα and granzyme B expression by T, NKT-like and NK cells are shown in Table 2. The inhibitory effect of 10^-6^ M methylprednisolone on IFNγ and TNFα expression by NK cells was greater than for T and NKT-like cells. In the presence of 2.5 ng/mL Cyclosporin A there was a significant inhibition of IFNγ and TNFα expression by all subsets. The combination of 10^-6^ M methylprednisolone and 2.5 ng/mL Cyclosporin A resulted in a synergistic inhibition of IFNγ and TNFα expression by T and NKT-like cells. Representative plots showing the combined inhibitory effect of 10^-6^ M methylprednisolone and 2.5 ng/mL Cyclosporin A on IFNγ expression by Pgp1+ T and NKT-like cells are shown in Figure 6. Higher concentrations of Cyclosporin A (>10 ng/mL) resulted in almost complete inhibition of IFNγ and TNFα expression by T, NKT-like and NK cells (data not shown). There was no inhibitory effect of 10^-6^ M methylprednisolone on granzyme B expression by T, NKT-like and NK cells. Significant inhibition of granzyme B expression by T, NKT-like and NK cells was only noted in the presence of 200 ng/mL and 250 ng/mL Cyclosporin A (e.g., 10 ± 4, 1 ± 2, 4 ± 3% inhibition of granzyme B in the presence of 200 ng/ml Cyclosporin A and 28 ± 8, 6 ± 4, 18 ± 6% inhibition of granzyme B in the presence of 250 ng/mL Cyclosporin A by T, NKT-like and NK cells respectively). The addition of 10^-6^ M methylprednisolone resulted in no additional effect on Pgp1 expression by any cell subset (data not shown). To determine the association of Pgp1 expression with drug resistance, the inhibitory effect of the various drug combinations on IFNγ and TNFα expression by Pgp1+ T, NKT-like and NK cells was investigated and results were almost identical to those for total IFNγ and TNFα expression by Pgp1+ T, NKT-like and NK cells (Table 3) and suggests a strong association between Ppg1 expression and drug resistance in T, NKT-like and NK cells.

**Table 2:** **The inhibitory effect of 10**^**-6**^ **M methylprednisolone (MP) alone and in combination with 2.5 ng/mL Cyclosporin A (CsA) on IFNγ and TNFα expression by T, NKT-like and NK cells are shown (mean ± SEM)**

		**IFNγ**			**TNFα**		
**Drug**	**Dose of drug**	**T**	**NKT**	**NK**	**T**	**NKT**	**NK**
**MP**	**10**^**-6**^ **M**	2 ± 2*	**5 ± 3**	**44 ± 8**	**2 ± 2**	**3 ± 2**	**47 ± 9**
**CsA**	**2.5 ng/ml**	**47 ± 9**	**44 ± 7**	**91 ± 5**	**51 ± 6**	**31 ± 9**	**92 ± 8**
**MP + CsA**	**10**^**-6**^ **M + 2.5 ng/ml**	**88 ± 7**	**83 ± 7**	**96 ± 6**	**81 ± 7**	**76 ± 7**	**96 ± 8**

The combination of 10^-6^ M methylprednisolone and 2.5 ng/mL Cyclosporin A resulted in the synergistic inhibition of IFNγ and TNFα expression by T and NKT-like cells.

* The percentage inhibition of IFNγ and TNFα by T, NKT-like and NK cells compared with control (same specimen) without drug/s.

**Table 3:** **The inhibitory effect of 10**^**-6**^ **M methylprednisolone (MP) alone and in combination with 2.5 ng/mL Cyclosporin A (CsA) on IFNγ and TNFα expression by Pgp1+ T, NKT-like and NK cells are shown (mean ± SEM)**

		**IFNγ**			**TNFα**		
**Drug**	**Dose**	**T**	**NKT**	**NK**	**T**	**NKT**	**NK**
**MP**	**10**^**-6**^ **M**	2 ± 2*	**4 ± 3**	**45 ± 9**	**3 ± 2**	**5 ± 2**	**49 ± 9**
**CsA**	**2.5 ng/ml**	**50 ± 10**	**46 ± 8**	**89 ± 7**	**54 ± 6**	**27 ± 6**	**87 ± 7**
**MP + CsA**	**10**^**-6**^ **M + 2.5 ng/ml**	**81 ± 5**	**76 ± 8**	**96 ± 7**	**78 ± 7**	**72 ± 8**	**97 ± 9**

The combination of 10^-6^ M methylprednisolone and 2.5 ng/mL Cyclosporin A resulted in the synergistic inhibition of IFNγ and TNFα expression by Pgp1+ T and NKT-like cells.

* The percentage inhibition of IFNγ and TNFα by Pgp1+ T, NKT-like and NK cells compared with control (same specimen) without drug/s.

## Discussion

This is the first study to show differential expression of the drug efflux pump Pgp1 by T, NKT-like and NK cells from COPD patients compared with healthy control subjects. COPD is a systemic disease [2] and we have previously shown increased IFNγ and TNFα by T cells [3], granzyme B by NK and NKT-like cells [5] and granzyme B by T cells [4] in the peripheral blood and lungs of COPD patients. Our novel findings that Pgp1 is up-regulated in NKT-like and NK cells in patients with COPD and that this is associated with increased pro-inflammatory and cytotoxic molecules in T, NKT-like and NK cells have important implications for treatment strategies to target these cells.

The relative lack of corticosteroid efficacy in COPD has been poorly understood and a major limiting factor in COPD treatment [2]. We now show that production of IFNγ and TNFα and granzyme B by T and NKT-like subsets of lymphocytes are not inhibited with therapeutic doses of methylprednisolone, a commonly used corticosteroid *in vitro*, confirming clinical findings. Importantly we show that by targeting Pgp1 with a low dose of the inhibitor, cyclosporine A, production of the pro-inflammatory cytokines IFNγ and TNFα are significantly inhibited. Further, a combination of very low dose cyclosporine A (2.5 ng/mL) with standard dose methylprednisolone (10^-6^ M), results in synergistic inhibition of these pro-inflammatory cytokines known to have systemic effects in patients with COPD [2]. The excellent negative correlation between efflux of Calcein-AM, previously shown to identify Pgp1 function in cells [8] and our findings of Pgp1 expression in T, NKT-like and NK cells confirms these novel findings.

Our group has undertaken pioneering work on the role of T-cell pro-inflammatory cytokines, particularly TNFα and IFNγ, and their role in COPD [3]. T cells are a major inflammatory cell type present in the lung in COPD patients. Our findings in 2007 were the first comprehensive report of intracellular pro- and anti-inflammatory T cell cytokines in the separate compartments of blood, bronchoalveolar lavage and intraepithelial T cells from bronchial brushings from COPD subjects and smokers. Interestingly, T-cell derived TNFα has been shown to cause apoptosis of airway epithelial cells and impair the clearance of these cells by alveolar macrophages [10]. Recently, TNFα has been described as the “driving force behind COPD” [11], and induction of TNFα in the lung has been shown to result in emphysema in the mouse model [9]. TNFα has also been shown to induce IL-2Rs and IFNγ production by T cells and activate neutrophils, macrophages, endothelial cells and fibroblasts [12]; cells that play important roles in the pathogenesis of COPD [2]. Recently it has been shown that fractalkine, a potent chemoattractant for monocytes and T cells produced by airway smooth muscle cells, was induced in the presence of both IFNγ and TNFα [13]. Furthermore, increased TNFα levels have been shown to be increased in diseases associated with COPD such as cardiovascular disease and as such, systemic treatment with low dose Cyclosporin A and prednisolone may result in improvements of a broad range of inflammatory conditions associated with COPD [14].

An important extension of this work would be to study T, NKT-like and NK cells in both the airways and lung tissue of COPD patients as we have previously done [5,15] to determine the role Pgp1 may play in steroid resistance in these compartments. If this hypothesis is correct, targeting the airways with inhaled low dose CsA combined with steroid may be the treatment of choice to inhibit these pro-inflammatory molecules associated with COPD disease.

It would also be of interest to study Pgp1 expression in lymphocyte subsets in the peripheral blood of smokers who have not progressed to COPD. Our previous findings of increased T-cell production of IFNγ and TNFα in the peripheral blood of COPD patients but not smokers without COPD suggests Pgp1 may not be upregulated in smokers who have not progressed to COPD. However, there may be a subset of susceptible smokers who do have increased Pgp1 in these cells who have an increased risk of developing COPD and further studies are warranted to investigate this hypothesis.

Our present findings show that there was a significant increase in Pgp1 expression by T and NKT-like cells compared with NK cells suggesting these subsets of lymphocytes may be the most resistant to effects of therapeutic drugs.

We showed that the cytotoxic molecule, granzyme B is unaltered by standard dose methylprednisolone and requires much higher concentrations of cyclosporine usually used for immunosuppression in patients such as those following lung transplantation [16]. Our results suggest that patients with high levels of this cytotoxic molecule may require treatment with higher dose Cyclosporin A. Further, identification of patients with high levels of granzyme B and response following treatment may allow tailoring therapeutics to individual patients using these techniques, to optimize immunosuppression as to possibly avoid problems associated with over-immunosuppression (e.g., infection and malignancy) and under-immunosuppression with worsening of COPD symptoms.

## Conclusion

In conclusion, COPD is associated with increased Pgp1 expression by peripheral blood T, NKT-like and NK cells co-expressing IFNγ, TNFα and granzyme B. Treatment strategies that target Pgp1 in T, NKT-like and NK cells may reduce systemic inflammatory mediators in COPD and improve patient morbidity.

## Competing interests

The authors declare that they have no competing interests.

## Authors’ contributions

GH performed the concept and design of experiments, analysis and interpretation of data and manuscript preparation; MH supplied and characterized patient specimens and helped draft the manuscript; HJ supplied and characterized patient specimens and helped draft the manuscript; PNR supplied and characterized patient specimens and helped draft the manuscript; SH helped with study design, statistical analysis and helped draft the manuscript. All authors read and approved the final manuscript.
